# AI-based body composition measures in abdominal CT scans: prime time for clinical implementation?

**DOI:** 10.1007/s00330-024-10936-9

**Published:** 2024-07-12

**Authors:** Lukas Müller

**Affiliations:** Department of Diagnostic and Interventional Radiology, University Medical Center of the Johannes Gutenberg-University Mainz, Mainz, Germany

Over the past few decades, several studies have shown that body computed tomography (CT) scans contain additional, reproducible, and objectively measurable volumetric information beyond the image data used for diagnosis [1]. This additional information includes body composition (BC) parameters such as muscle mass and density, visceral and subcutaneous fat, and liver fat. For a long time, manual and semi-automated measurements limited the application of BC assessment to the scientific field. However, with the rise of artificial intelligence (AI) in image analysis, AI-based automated quantification has acted as a catalyst, ensuring that the number of population-based and entity-specific studies on BC has significantly increased. The use of fully automated AI-based measurement of BC parameters has not only made the results reproducible but also enabled large-scale applications [2]. This has created the potential for everyday clinical use [3].

One aspect of BC assessment using abdominal CT scans is the determination of abdominal adipose tissue [1]. In addition to the so-called “typical” orthotopic adipose tissue (i.e., subcutaneous adipose tissue (SAT)), ectopic adipose tissue can also be determined. Ectopic adipose tissue refers to fat deposition in locations not typically associated with adipose tissue (i.e., visceral adipose tissue (VAT) or liver fat). Both orthotopic and ectopic adipose tissues are associated with metabolic dysregulation [4, 5].

In this issue of *European Radiology*, Lee et al [6] focus on these abdominal tissue components, which both are associated with obesity. Obesity is an increasingly prevalent condition with a high socioeconomic burden and a strong association with all-cause mortality and cardiometabolic disease [7, 8]. However, cardiovascular health and risk in patients with obesity are not fully assessed by conventional measurement techniques such as BMI, body weight, or waist circumference [8]. In addition, these methods cannot differentiate between different abdominal tissue types. In comparison, CT-based BC measurements allow differentiation and quantification of various adipose tissue types, enabling a deeper analysis of their associations with cardiometabolic risk.

This is particularly important when patients undergo a CT scan for another reason, and this information becomes additionally available. In their study, Lee et al evaluated the potential of fully automated CT-based measurements of different abdominal adipose tissues in predicting all-cause mortality, future cardiovascular disease, and diabetes in a cohort of more than 9000 asymptomatic adults undergoing CT colonography screening [6]. In this cohort, higher measures of VAT, visceral-to-subcutaneous adipose tissue ratio (VSR), liver fat, and muscle fat were associated with increased mortality, cardiovascular events, and diabetes risk.

An important new aspect of the study is that it shows the complex relationship between different abdominal fat depots, which cannot be captured by conventional measurement methods (e.g., BMI). While abdominal tissue measurements outperformed BMI in predicting mortality risk, two significant findings should be highlighted: (1) VSR as a composite parameter is a stronger predictor of mortality and cardiovascular events than VAT and SAT alone; (2) for the first time, a “U-shaped” relationship between SAT area and all-cause mortality could be demonstrated, correlating with the so-called “obesity paradox” previously observed only for BMI. Notably, this study also confirmed the strong correlation between low muscle attenuation (i.e., myosteatosis) and mortality risk. The results of this study emphasize that the knowledge of BC and its importance as a value-added biomarker for mortality and cardiometabolic risk in general, but also for disease-specific mortality, continues to grow. Especially with the possibilities of AI-based, fully automated determination of BC parameters, we have an incredibly valuable and powerful tool at our disposal to obtain additional image-based patient information.

However, this data also needs to be used and properly evaluated. Only when appropriate cut-off values and recommendations based on these values are available, BC parameters can compete with established clinical and anthropometric parameters as additional screening tools. Obviously, as confirmed in the study by Lee et al, they provide us with better predictive capabilities than, for example, BMI [6]. However, the main reason why BC parameters are not already included in every report is the lack of clarity in the radiology community on how they should be used and in what form they should be communicated with the clinical referral. It is no longer a question of proving that this additional information adds value, nor is it a question of the high complexity of measuring the BC parameters (especially with reliable algorithms and established pipelines). Rather, it is a matter of translating and exploiting the knowledge gained into applicable conditions. Clear reporting criteria are needed. These and related recommendations should be developed and validated jointly by radiologists and referring clinicians, similar to the RADS (Reporting and Data Systems) established for various types of reports [9]. An important aspect to mention here is that we, as radiology departments, are the producers of this information. As radiologists, we act as an important control center and gatekeeper for the correct interpretation of CT-based BC parameters.

Apart from standardization, other concerns currently hindering deep implementation in daily clinical routines include regulatory approval, commercial availability, reimbursement issues, and a lack of user-friendliness [2, 3]. In addition, standardization and acquisition of representative volumes for other imaging modalities for BC assessment remains a challenge for further studies.

In conclusion, the study by Lee et al emphasizes that BC assessment provides important additional information regarding the complexity of abdominal adipose tissue correlations and prognostic ability of abdominal fat. Fully automated AI-based tools offer the opportunity to utilize currently unused image data as value-added biomarkers. However, for the next step towards application in clinical routine, clear recommendations are needed on how to interpret and communicate BC parameters. As radiologists, we have an obligation to be at the forefront of developing and establishing such recommendations and application criteria.
